# Diabetic Ketoacidosis in Pregnancy: A Systematic Review of the Reported Cases

**DOI:** 10.1177/11795514241312849

**Published:** 2025-01-15

**Authors:** Dimitra Stathi, Florence Ning Lee, Mili Dhar, Stergios Bobotis, Elisavet Arsenaki, Taruna Agrawal, Konstantinos Katsikas Triantafyllidis, Konstantinos S Kechagias

**Affiliations:** 1 Department of Endocrinology and Diabetes, King’s College Hospital NHS Trust, London, UK; 2 Department of Endocrinology and Diabetes, St Bartholomew’s Hospital, London, UK; 3 Department of Metabolism, Digestion and Reproduction, Faculty of Medicine, Imperial College London, UK; 4 Department of Obstetrics and Gynaecology, The Hillingdon Hospitals NHS Foundation Trust, Uxbridge, UK; 5 Department of Nutrition and Dietetics, Royal Marsden Hospital, London, UK

**Keywords:** Diabetes, pregnancy, gestation, DKA, diabetic ketoacidosis

## Abstract

**Background:**

Diabetic ketoacidosis (DKA) is a rare but serious complication that can develop during pregnancy, with up to 30% of patients presenting with euglycemia, making prompt recognition challenging. It is associated with increased perinatal mortality rates, although the exact risk of maternal mortality remains unclear. The purpose of this systematic review was to examine the available literature and provide an overview of reported cases of DKA during pregnancy.

**Methods:**

PubMed, Web of Science and Scopus library databases were screened from inception until January 2024. Included studies provided data on classic or euglycemic DKA during pregnancy. All study designs were considered eligible for inclusion.

**Results:**

We identified 66 eligible articles, which included 57 case reports and case series with individual patient data, and 9 studies without individual patient data. The mean age at diagnosis was 28.8 years, and the average gestational age at diagnosis was 29.5 weeks. The majority of women had type 1 diabetes mellitus (T1DM) (45.9%), followed by gestational diabetes (GDM) (40.5%). Most cases were classified as classic DKA (70.3%), with nearly one-third developing euglycemic DKA (29.7%). The most common trigger factors were infections (28%), followed by poor adherence to treatment (13.5%). The most frequent symptoms included nausea (32.4%), vomiting (32.4%), osmotic symptoms (21.6%), and abdominal pain (20.2%). All cases were treated with intravenous insulin and fluids. The vast majority (98.9%) of women eventually fully recovered, with only 1 reported death due to organ failure (1.3%). Intrauterine death or stillbirth occurred in one-third of cases (35.2%), including 1 instance of a twin pregnancy.

**Conclusions:**

DKA is a condition that clinicians may encounter during pregnancy. Although rare, increased awareness and early recognition are crucial for optimal management and improved maternal and neonatal outcomes.

## Abstract (plain language summary)

Plain language summary

Understanding Diabetic Ketoacidosis (DKA) in Pregnancy: Causes, Risks, and the Importance of Early Treatment

Diabetic ketoacidosis (DKA) is a serious health issue that can affect pregnant women with diabetes. It happens when the body cannot use sugar properly because there isn’t enough insulin. As a result, the body breaks down fat for energy, which produces harmful substances called ketones. These ketones can build up and cause dangerous complications for both the mother and her baby. While DKA isn’t common, it can be life-threatening if not treated quickly.

This study looked at 66 reports about DKA in pregnancy to better understand how often it happens, what causes it, how it is treated, and what effects it has on the mother and baby. The study found that DKA mostly occurs in women with certain types of diabetes (type 1 or gestational diabetes) and typically happens later in pregnancy, especially during the third trimester.

The main reasons for DKA during pregnancy include infections, not following diabetes treatment properly, and taking certain medications like steroids. Common signs of DKA include feeling nauseous, vomiting, feeling very thirsty, needing to urinate often, and stomach pain. Most of the women in the study recovered after receiving treatment, which usually involved giving insulin and fluids through a drip. However, DKA was associated with a high rate of stillbirth, affecting about one-third of pregnancies.

This study highlights how important it is to recognise and treat DKA early during pregnancy. It also shows the need for doctors to closely monitor pregnant women with diabetes, especially if they are sick or on certain medications. Early action can help prevent serious complications and improve the chances of a healthy outcome for both mother and baby.

## Introduction

Diabetic ketoacidosis (DKA) is the most common emergency associated with diabetes. It is characterised by metabolic acidosis (bicarbonate concentration ⩽ 18.0 mmol/l and/or venous pH ⩽ 7.3), capillary or blood ketone concentration ⩾ 3.0 mmol/l or significant ketonuria (2+ or more on standard urine sticks) and in the classic presentation, a blood glucose concentration of 11.1 mmol/l or higher, versus normal plasma glucose or a milder hyperglycaemia (11-14 mmol/l) in the euglycemic DKA (EDKA).^1,2^ Pathophysiologically, the core of DKA is increased gluconeogenesis and glycogenolysis along with reduced glucose oxidation resulting from decreased glucose uptake by the peripheral tissues. These all lead to hyperglycaemia and subsequently osmotic diuresis and dehydration; furthermore, the increased free fatty acids are oxidised into ketone bodies in the liver resulting in a high anion gap metabolic acidosis.

The metabolic changes during pregnancy can lead to the rapid development of DKA even with relatively mild hyperglycaemia, although the exact mechanisms are not entirely understood.^3^ Despite this, DKA is rare in pregnancy, with an estimated incidence of 0.5% to 3%.^4^ Up to 30% of cases are euglycemic, making prompt recognition more challenging.^5^ Although DKA is not commonly life threatening to the pregnant woman if it is recognised and treated promptly, the exact risk of maternal mortality secondary to complications associated with DKA is not well established.^6^ Perinatal mortality rates, on the other hand, remain as high as 35% for a single episode of DKA despite substantial improvements in perinatal and neonatal care.^3^ Therefore, raised awareness is required for an immediate diagnosis and management.

The purpose of this systematic review is to examine the available literature, provide an overview of the reported cases of DKA in pregnancy and elaborate on the diagnosis, management and associated outcomes.

## Methods

This review was reported based on the ‘Preferred Reporting Items for Systematic Reviews and Meta-Analyses’ (PRISMA) guidelines.^7^

### Literature search

Two reviewers (DS, KKT) searched the PubMed, Web of Science and Scopus library databases from inception until January 2024. The search included the following terms: (diabetic ketoacidosis OR DKA OR diabetic acidosis OR Diabetic ketosis) AND (pregnancy OR gestation). There were no restrictions regarding study design or geographic region. A manual search of references cited in the selected articles was also conducted for undetected studies. Discrepancies in the literature search process were resolved by a third investigator (KSK).

### Eligibility criteria

Included studies provided data on classic or EDKA during pregnancy. All study designs were considered eligible for inclusion. Only articles with full text in English language were included. Cases on the immediate postpartum period were excluded. Review articles, abstracts submitted in conferences and non-peer reviewed sources were also considered ineligible for inclusion.

### Data extraction and handling

In all articles, patient data was collected and handled by 3 authors (DS, FNL, MD) who conducted the data extraction independently. The following information was collected: article type, year, type of diabetes, age, ethnicity, parity, week of gestation, type of DKA (classic or euglycemic), medical history, regular medications, trigger factors, presenting symptoms, laboratory results, treatment, maternal outcomes, foetal outcomes including preterm delivery, growth and presence of oligohydramnios, mode of delivery (spontaneous vaginal birth, operative vaginal delivery, emergency or elective caesarean section) and type of labour (spontaneous or induced). Any disagreements were discussed and resolved by a fourth author (KSK).

### Quality assessment

The risk of bias (RoB) for studies with individual patient data (case reports and series) was independently assessed by 2 authors (KSK and KKT). To evaluate the overall quality of these studies, the critical appraisal checklist provided by the Joanna Briggs Institute (JBI) was employed. The assessment was based on the reporting of 8 elements: patient demographics, medical history, health status, physical examination and diagnosis, concomitant therapies, post-intervention health status, drug administration, and reaction interface. Each element was scored as ‘Yes’, ‘No’, ‘Unclear’ or ‘Not Applicable’, depending on the availability of information.^8^

For studies without individual patient data (observational studies), the risk of bias was assessed using the Methodological Index for Non-Randomised Studies (MINORS). MINORS is a validated tool designed to evaluate the methodological quality of non-randomised studies, whether comparative or non-comparative. Each domain within MINORS is scored as 0 if not reported, 1 if reported with inadequate details, and 2 if adequately reported. The global ideal score is 16 for non-comparative studies. Studies with MINORS scores < 6 were considered to have a high risk of bias, while those with scores between 6 and 9 were considered to have a moderate risk of bias.^9^

### Data synthesis

We used descriptive statistics to describe the demographics and clinical characteristics of the included patients. Means for continuous variables and frequencies and percentages for binary variables were used. We reported duration of symptoms in days due to inconsistent reporting of this information in the included articles.

## Results

### Literature search and study characteristics

**Figure 1.**
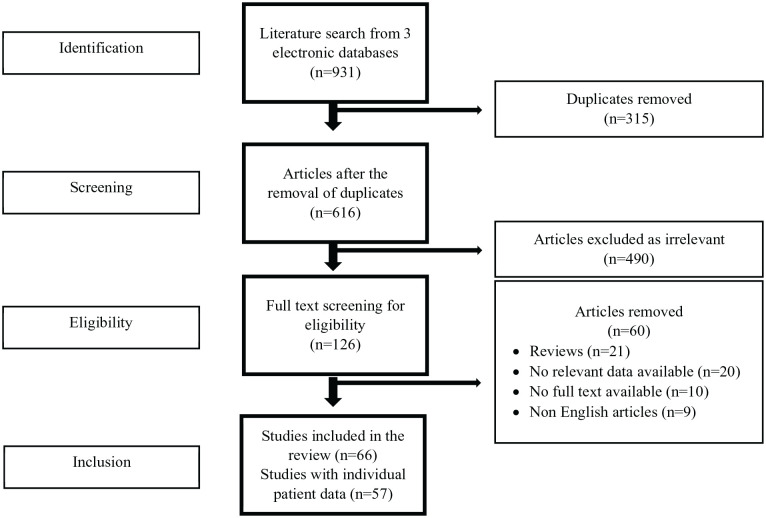
PRISMA flowchart.

The initial literature search yielded 931 publications. In the first screening 490 studies were excluded as irrelevant. After the exclusion phase, 126 full texts were screened and 66 studies were found eligible for the systematic review (Figure 1).^4,10-65^ Of the studies 20 were conducted in Asia, 20 in Americas, 15 in Europe, 1 in Africa and 1 in Oceania. Regarding study design, 9 studies were case series comprising at least 3 cases each without individual patient data, and 57 were case reports and case series with individual patient data (Tables 1 and 2).

**Table 1.** Characteristics of the included studies (studies with individual patient data).

Author	Case number	Age	Gestational age at diagnosis and parity	Type of Diabetes and DKA	Trigger factor	Presenting symptoms	Treatment	Maternal outcomes	Foetal outcomes
Year									
Country									
Ali	Case 1	Mid 20s	31 + 5 wk	T1DM, classic DKA	Long haul travel, dehydration	Bilateral ankle swelling, decreased foetal movements, vomiting	IV fluids and insulin	Fully recovered	Live birth at 37 wk with ventouse
2023									
UK									
Stamatiades	Case 1	32	27 wk	Newly diagnosed T1DM, classic DKA	Covid-19	Shortness of breath, congestion, loss of taste and smell, polyuria, polydipsia	IV fluids and insulin	Fully recovered and started on MDI	NA
2023									
USA									
Muppidi	Case 1	23	37 wk	T1DM, EDKA	UTI, low caloric intake	Nausea, vomiting	IV fluids, high dose sliding scale insulin, empiric ceftriaxone	Fully recovered	No complications
2020									
USA									
Yaron	Case 1	35	29 wk G2P1	T1DM, EDKA	Severe carbohydrate restriction	Severe nausea, vomiting, tachypnoea, abdominal pain	IV fluids and insulin	Fully recovered but readmitted with DKA due to carb restriction	No complications
2020									
Israel									
Jaber	Case 1	30	32 + 3 wk G2P1	T1DM, EDKA	No specific trigger factor identified	Nausea, vomiting, Kussmaul breathing	Insulin infusion, bolus IV fluids, a maintenance fluid rate of 5% dextrose/0.45% NaCl solution	Intubation and mechanical ventilation, then extubated on day 2, and fully recovered	Intrauterine foetal demise
2019									
USA									
de Alencar	Case 1	38	34 wk G2P0	T1DM, EDKA	Nonadherence to insulin	Nausea, vomiting, anorexia, abdominal pain	IV fluids and insulin	Fully recovered	Delivered via emergency c-section due to bradycardia
2019									
Brazil									
Hayakawa	Case 1	31	30 + 6 wk G1P1	Fulminant T1DM, classic DKA	Coxsackievirus B	Fatigue, SOB	IV fluids and insulin	Fully recovered and discharged on MDI	Still birth – delivered via emergency c-section
2019									
Japan									
Wazir	Case 1	35	29 wk G3P2	GDM, EDKA	Covid-19	Fever, cough, SOB, nausea, vomiting, loss of appetite, diarrhoea	NA	Fully recovered	Delivered without complications at 40 wk
2022									
Qatar									
Velasco	Case 1	29	34 wk	GDM, EDKA	Covid-19	NA	IV insulin and fluids, oxygen therapy, remdesivir	Recovered, but due to persistent hypoglycaemia	Neonatal hypoglycaemia, delivered at 36 wk via CS
2022									
Spain									
Bereda	Case 1	28	31 wk G2P2	T1DM, classic DKA	No specific trigger factor identified	Nausea, vomiting	IV insulin and fluids	Fully recovered	NA
2022									
Ethiopia									
Harman	Case 1	32	28 + 3 wk G2P0	GDM, EDKA	Covid-19	Fatigue, congestion, SOB, wheezing, chest tightness	IV fluids and insulin, albuterol, dexamethasone, Lovenox	Mechanical ventilation due to severe hypoxia	Delivered via emergency CS and intubated
2022									
USA									
Dargel	Case 1	23	27 wk G2P0	T1DM, EDKA	Starvation, hyperemesis	Abdominal pain, vomiting	IV fluids and insulin	Fully recovered	Still birth
2021									
Germany									
Smati	Case 1	36	32 wk G9P7	GDM, EDKA	Covid-19	Abdominal pain, nausea, vomiting, SOB	IV fluids and insulin	Fully recovered	Delivered via emergency CS, no complications
2021									
France									
Aminimoghaddam	Case 1	27	28 wk G3P1	Insulin dependent diabetes, classic DKA	Covid-19 and poor compliance to treatment	SOB, nausea, dry cough, reduced foetal movements	IV fluids and insulin, antibiotics	ARDS, organ failure, death	IUFD
2021									
Iran									
Lucero	Case 1	22	Early pregnancy confirmed during admission	T1DM, EDKA	NA	Vomiting and abdominal pain	IV fluids and insulin	Fully recovered	NA
2018									
Argentina									
Eskoli	Case 1 and 2 (readmission)	31	28 wk G8P3	T2DM, EDKA	Poor adherence to treatment	Recurrent vomiting	IV fluids and insulin	Discharged and represented at 29 wk with abdominal pain and vaginal bleeding; again on DKA	significant metabolic acidosis, convulsions, severe brain damage, respiratory failure, death
2021	Case 3	24	24 wk G3P2	T1DM, classic DKA	New diagnosis	Blurred vision	IV fluids and insulin	Fully recovered and started on MDI and later on CSII	Delivered without complications at 38 wk
Israel	Case 4	31	Pregnancy confirmed during admission G2P1	T1DM, classic DKA	CSII failure	2 D delay of menstruation	IV fluids and insulin	Fully recovered	Diagnosed with a missed abortion at 10 wk of gestation
	Case 5 and 6	23	34 wk G5P1	T1DM, classic DKA	CSII failure and poor adherence	Urinary urgency, dysuria, vomiting	IV fluids and insulin	Fully recovered; represented during her next pregnancy with DKA and similar trigger factors	NA
	Case 7	28	30 wk G2P1	T2DM, classic DKA	Infection	Recurrent vomiting for a week and a half	IV fluids and insulin	Fully recovered	Delivered via planned CS at 37 wk
	Case 8	29	34 wk G3P1	T1DM, classic DKA	Poor adherence	Recurrent vomiting	IV fluids and insulin	Fully recovered	Delivered via planned CS at 36 wk
Farrant	Case 1	36	36 wk	Fulminant T1DM, classic DKA	NA	SOB, lower abdominal pain, polydipsia	IV fluids and insulin	Fully recovered	Respiratory distress, hyperglycaemia, hypoxic, ventilation
2016	Case 2	42	37 wk	Fulminant T1DM, classic DKA	ΝΑ	Thirst, lower abdominal pain and decreased foetal movements	IV fluids and insulin	Fully recovered	Hyperglycaemia
New Zealand									
Cardonell	Case 1	25	35 + 4 wk G3P2	T2DM, EDKA	NA	Severe abdominal pain, headache, visual changes	IV fluids and insulin	Respiratory distress requiring NIV	Delivered via emergency CS, atrial and ventricular septal defects
2016									
USA									
Cen	Case 1	31	28 wk	Fulminant T1DM, classic DKA	NA	Loss of appetite, fatigue, chest discomfort, SOB, polyuria, polydipsia	IV fluids and insulin	NA	stillbirth
2014	Case 2	17	37 wk	Fulminant T1DM, classic DKA					
China									
Himuro	Case 1	32	28 wk G2P1	LADA, classic DKA	NA	Generalised fatigue, thirst, ketotic odour	IV fluids and insulin	Fully recovered	Delivered full-term via vaginal birth
2014									
Japan									
Graham	Case 1	30	30 wk G4P1	GDM, EDKA	Poor adherence to insulin therapy	Vomiting	IV fluids and insulin	Fully recovered	Delivered at 36 wk with CS. No complications
2014									
UK									
Melville	Case 1	31	30 + 6 wk G5P4	T2DM, classic DKA	Not on treatment	2-D history of malaise and weakness, polydipsia, polyuria and weight loss.	Insulin sliding scale	Fully recovered	intrauterine death, polyhydramnios and transverse lie of foetus, Postmortem reported a macrosomic male infant weighing 1890 with established neuronal hypoxic ischaemic encephalopathy
2014									
UK									
Dorey	Case 1	20	28 wk	T1DM, classic DKA	Steroids for foetal lung maturation	Hyperglycaemia noted on hospital monitoring	IV insulin	Pre-eclampsia x2 cases	LGA delivered at 28 wk, polyhydramnios
2014	Case 2	29	28 wk						PROM at 29 wk, 34th percentile size, polyhydramnios
France	Case 3	25	NA						SGA delivered at 31 wk
Yan	Case 1	23	32 wk	Fulminant T1DM, classic DKA	Respiratory infection	Nausea, vomiting, polyuria, fever, excessive thirst, reduced foetal movements	IV insulin, fluids and antibiotics	Fully recovered	Stillbirth
2013	Case 2	21	25 wk						
China									
Kim	Case 1	31	19 wk	Fulminant T1DM, classic DKA	NA	Nausea, vomiting, headache, dizziness	IV insulin and fluids	Fully recovered	Delivered full term with vaginal birth
2012									
Korea									
Dharbamulla	Case 1	30	33 wk G1P0	GDM, EDKA	Sepsis/UTI	Coffee ground vomting, epigastric pain	IV insulin and fluids	Fully recovered	LGA, delivered full-term
2012									
UK									
Napoli	Case 1	26	34 wk G3P2	T1DM, EDKA	Insulin omission/eating disorder, self-induced vomiting	Vomiting	IV insulin and fluids	Fully recovered	neonatal metabolic acidosis and respiratory distress, electrolyte imbalance, LGA
2011									
Italy									
Pinto	Case 1	33	33 wk G1P0	GDM, classic DKA	sepsis	Polyuria, polydipsia, Kussmaul’s breathing	IV insulin and fluids	Fully recovered	Intrauterine death
2011	Case 2	21	29 wk G2P1	GDM, classic DKA	UTI				
Peru									
Lee	Case 1	28	33 wk G1P0	Fulminant T1DM, classic DKA	NA	Polyuria, polydipsia, abdominal pain, SOB, poor apetite	NA	Fully recovered	Stillbirth
2010									
Taiwan									
Tan	Case 1	28	18 wk G1P0	Fulminant T1DM, classic DKA	NA	Abdo pain, Nausea, vomiting, polyuria, polydipsia	IV fluids and insulin	Fully recovered	Stillbirth
2010									
Malaysia									
Franke	Case 1	23	32 wk G1P0	GDM, EDKA	Starvation ketosis, occult sepsis, recent influenza A infection	5-D history of flu-like symptoms, nausea and vomiting	IV antibioticsinsulin and fluids	Fully recovered and discharged on MDI, which was discontinued 5 wk later	Delivered at 38 wk of gestation
2009									
UK									
Chico	Case 1	29	34 wk G4P0	T1DM, classic DKA	Labour	No other symptoms, DKA diagnosed during labour	IV fluids and insulin	Fully recovered	Delivered pre-term via c-section
2008									
USA									
Tarif	Case 1	37	35 wk	T2DM, EDKA	Insulin omission	Nausea, abdo pain	IV fluids and insulin	Fully recovered	No complications
2007									
Saudi Arabia									
Oliver	Case 1	29	28 wk G2P1	T2DM, EDKA	Infection/bronchopneumonia	Hyperventilating, sore throat, reduced oral intake	IV fluids and insulin	Fully recovered	Delivered pre-term without any complications
2007									
UK									
Yamamoto	Case 1	28	36 wk G4P3	Fulminant T1DM, classic DKA	NA	Nausea, vomiting, flu-like symptoms, fatigue, Kussmaul breathing	IV fluids and insulin	Fully recovered	Stillbirth, oligohydramnios
2007									
Japan									
Kamalakannan	Case 1	28	36 wk G4P3	T1DM, classic DKA	Insulin omission	Hyperventilating, dehydrated	IV fluids and insulin	Fully recovered	Stillbirth
2003									
UK									
Inagaki	Case 1	37	19 wk G2P1	T1DM, classic DKA	NA	Nausea, abdominal fullness, excessive thirst	IV fluids and insulin	Fully recovered	Stillbirth
2002									
Japan									
Deepak	Case 1	23	31 wk G1P0	GDM, EDKA	Alcohol abuse, urinary tract infection	3-D history of nausea, persistent vomiting, abdominal pain	IV fluids and insulin	Fully recovered	Delivered at 40 wk gestation weighing 3.24 kg
2002									
UK									
Trivedi	Case 1	32	28 wk G2P1	GDM, classic DKA	Rhino cerebral mucormycosis	Fever and tachypnoea	IV insulin, IV Amphoterin B	Fully recovered	Delivered at 34 wk gestation weighing 1.8 kg
2002									
Mumbai									
O’Shaughnessy	Case 1	23	36 wk G2P1	GDM, classic DKA	NA	Contractions every 3 min, decreased foetal movement, polyuria and polydipsia.	IV fluids and insulin	Fully recovered	foetal late decelerations and oligohydramnios
1999									
USA									
Sasuga	Case 1	28	33 wk	GDM, classic DKA	NA	Polyuria, polydipsia, lethargy, dyspnoea	IV fluids and insulin	Fully recovered	Delivered via CS, intubated and ventilated; intracranial haemorrhage, now living with cerebral palsy and cognitive impairment
1999									
Japan									
Pitteloud	Case 1	25	32 wk G1P0	GDM, classic DKA	Newly diagnosed diabetes	2-D history of vomiting, vertigo, polydypsia, and polyuria	IV insulin and fluids	Fully recovered	Macrosomia, delivered full-term
1998									
USA									
Bedalov	Case 1	40	32 wk Multiparous	GDM, EDKA	Administration of glucocorticoid, prolonged fast	Nausea, vomiting, tachypnoea	IV fluids and insulin	Fully recovered	NA
1997									
USA									
Ko1995Hong Kong	Case 1	26	33 wk G1P0	GDM, classic DKA	Infection	Decreased foetal movements for 1 d, polyuria, polydipsia, chills, malaise and nausea for 3 d	IV fluids, insulin and antibiotics	Fully recovered and discharged on MDI	Stillbirth
	Case 2	28	38 wk G2P1	GDM, classic DKA	Infection	Abdominal pain, breathlessness and drowsiness	IV fluids, insulin and antibiotics	Fully recovered and discharged on MDI	Stillbirth
Sills	Case 1	14.5	35 wk	Insulin dependent diabetes, classic DKA	NA	Leg muscle aches, lower abdominal pain, blurry vision, no foetal movements for 24 h, increased thirst and increased urination	IV fluids, insulin and magnesium sulphate	She was additionally diagnosed with preeclampsia. Fully recovered.	Stillbirth
1994									
USA									
Abourizk	Case 1	34	20 wk G3P2	GDM, classic DKA	NA	Vaginal bleeding, ruptured membrane, polyuria, polydipsia, polyphagia, nocturia.	IV fluids and insulin	Fully recovered	Stillborn
1993									
USA									
Lindenbaum	Case 1	36	29 wk G3P2	T1DM, classic DKA	Pump failure	Nausea, vomiting, disorientation	IV fluids and insulin	Fully recovered	Healthy baby was born at 37 wk gestation
1993									
USA									
Maislos	Case 1	40	30-32 wk G11P10	GDM, classic DKA	UTI	Fever, dysuria, SOB	IV fluids, insulin, antibiotics	Fully recovered	Stillbirth, polyhydramnios
1992									
Israel									
Clark	Case 1	34	36 wk G3P1	GDM, EDKA	Respiratory infection	Sore throat, fever, SOB, nausea, vomiting	IV insulin and fluids	Fully recovered	Live macrosomic baby delivered via CS
1991									
UK									
Bertolino	Case 1	18	8.7 wk G1P0	T1DM, classic DKA	Gastroenteritis and insulin omission	Nausea, vomiting, abdominal cramping, loose stools	IV insulin and fluids	Fully recovered	Elective termination of pregnancy
1990									
USA									
Bernstein	Case 1	20	28 wk	GDM, classic DKA	Administration of betamethsone and terbutaline for preterm labour	Preterm	IV insulin and fluids	Fully recovered but readmitted 9 d later with PROM	Infant born at 30 wk, respiratory distress, intraventricular haemorrhage, polyhydramnios
1990									
USA									
Halpren	Case 1	27	32 wk G2P1	GDM, classic DKA	UTI	Flank pain, nausea, vomiting, dryness of mouth with polydipsia, polyuria	IV fluids, insulin and ritodrine	Fully recovered	Neonatal hypoglycaemia and hypocalcaemia
1988									
USA									
Robertson	Case 1	29	29 wk G2P0	GDM, classic DKA	NA	3-D history of nausea, vomiting, polydipsia and polyuria	IV fluids and insulin	Fully recovered but required long term insulin	Delivered at 38 wk, no complications
1986									
UK									
Rhodes	Case 1	23	30 wk G1P0	T1DM, Classic DKA	Respiratory tract infection	SOB, nausea, vomiting, tachypnoea, moderate thirst	IV fluids and insulin	Fully recovered	Delivered a healthy baby at 36.5 wk
1984									
USA									
Knowles	Case 1	25	25 wk G4P3	GDM, classic DKA	Dental abscess	polydipsia, polyuria	IV fluids, insulin and antibiotics	Fully recovered from DKA, but complicated by stitch abscess and tubo-ovarian abscess; had a total hysterectomy	NA
1962									
USA									
Phuapradit	Case 1	31	31 wk G1P0	GDM, classic DKA	Severe preeclampsia	Dyspnoea	IV insulin and fluids	Fully recovered	Macerated foetus weighing 1750 g
1993	Case 2	21	35 wk G1P0		NA	Dyspnoea			Macerated male foetus weighing 3500 g
Thailand	Case 3	32	30 wk G2P1		Given dexamethasone, terbutaline for preterm labour	Preterm labour			Live female foetus weighing 3500 g
	Case 4	38	34 wk G2P1		Given dexamethasone, terbutaline for preterm labour	Antepartum haemorrhage and preterm labour			Macerated female foetus weighing 2,400 g
	Case 5	29	30 wk G3P1		Given dexamethasone, terbutaline for preterm labour	Antepartum haemorrhage and preterm labour			Live male infant weighing 3800 g
Otsubo2002Japan	Case 1	37	29 wk G2P1	T1DM, classic DKA	Chorioamnionitis	Slight uterine contractions, thirst, vomiting, and general fatigue	IV fluids and insulin	Fully recovered	Stillbirth
	Case 2	36	19 wk G2P1	T1DM, classic DKA	Viral infection	Polyuria, polydipsia, general fatigue, vomiting, and abdominal tenderness	IV fluids and insulin	Fully recovered	Stillbirth

**Table 2.** Characteristics of the included studies (studies without individual patient data).

Author	No of cases, age	Parity and gestational age at diagnosis	Type of diabetes and DKA	Trigger factor	Presenting symptoms	Treatment	Maternal outcomes	Foetal outcomes
Year								
Country								
Schneider	4 Cases, 25 ± 1	29 ± 1 wk	GDM	Infection (27%), history of omission of insulin therapy (18%)	NA	IV insulin	No maternal deaths. Insulin therapy was stopped in all cases	One intrauterine foetal demise, 3 cases reached full-term pregnancy
2003								
USA								
Cullen	11 Cases, n/a	8 to 37 wk with a mean of 26 wk	Pre-existing diabetes (type 1/2), classic DKA	NA	Nausea and vomiting	8 Patients treated with low-dose intravenous insulin, 3 patients with SC insulin. All patients received IV fluids	Two patients required CS for foetal distress.	One foetal death in a patient with severe acidosis at 27 wk; Of the 10 term infants, the mean birthweight was 3812 ± 592 g; 4 macrosomic infants
1996								
USA								
Kilvert1993UK	10 Cases, 12 to 49	2 wk (conception) to 36 wk, median 26 wk	9 With pre-existing diabetes, 1 with gestational diabetes	• Ritodrine infusion	• Case 1 (pyrexia and sore throat)	IV insulin	Six of the eight patients treated with insulin at the time of the ketoacidosis had, between them, seven further episodes of ketoacidosis and at least eleven admissions for severe hyperglycaemia either before or after the index event.	There was one intrauterine death at 27 wk gestation and one spontaneous abortion 2 wk after the ketoacidosis. The remaining seven foetuses survived, one was delivered by CS because of foetal distress.
				• Stopping insulin during an episode of gastroenteritis	• Case 2 (none)			
				• Infection	• Case 4 (coryza)			
				• Failure to diagnose gestational diabetes resulting in DKA	• Case 5 (ketoacidosis after omitting insulin while having gastroenteritis)			
					• Case 6 (premature labour, ketoacidosis post ritodrine infusion)			
					• Case 7 (hyperglycaemia followed by ketoacidosis			
					• Case 8 (reducing insulin during upper respiratory tract infection) case 9 (3 d history of air hunger)			
Dhanasekaran2022USA	71 Cases, 28.0 (22.3-33.0)	30.0 (18.4-33.0) wk	T1DM (82.8%) T2DM (17.2%)	• Steroid administration 5 (7.0)	NA	• No maternal deaths	NA	• Foetal demise occurred in 10 (17.2%) pregnancies (6 miscarriages and 4 stillbirths)
				• Device failure 5 (7.0)		• Preeclampsia 29.3%		• LGA (n = 16, 33.3%)
				• Preeclampsia 1 (1.4)		• 32.7% gestational hypertension		• Neonatal hypoglycaemia (n = 29, 60.4%)
				• Drug abuse 1 (1.4)				• Intensive care unit admission (n = 25, 52.1%).
				• Severe migraine 1 (1.4)				• Shoulder dystocia 4 (8.3%)
				• Infection 16 (22.5)				• Congenital anomaly 5 (10.4%).
				• Nonadherence 28 (39.4)				
				• New diabetes diagnosis 7 (9.8)				
Liu	30 Cases, 27.37 ± 4.62 y	191.65 ± 11.67 d	Fulminant T1DM	NA	Polydipsia, polyuria, disturbance of consciousness, flu-like symptoms, and gastrointestinal symptoms	No fatalities		Foetal demise occurred in 21 out of the 23 cases (91.3%)
2018								
China								
Bryant	33 Cases, 25 ± 5 y	17(5–34) weeks, multiparous 70%, nulliparous 30%	T1DM (67%); White’s classification A (gestational) 1, B 9 (27%), C 8 (35%), 10 (30%), R/F 5 (15%)	>50% poor compliance with insulin, 33% infection	Nausea and vomiting (97%)	Insulin given by 24 h: 87 ± 9.5, initial fluid resuscitation first 8 h: 3723 ± 223	8 Delivered during admission; 5 had severe pre-eclampsia	3 Infants admitted to the neonatal ICU > 24 h; 3 spontaneous abortions 0 stillbirths; 3 major malformations; 3 jaundice; 2 hypoglycaemia;3 major foetal malformation; 1 multiple anomalies; 2 congenital heart malformation; birth weight 2748 ± 180; 35% preterm birth.
2017								
USA								
Maseko	56 Cases, 29.6 y (range 20-43 y)	-	36 (64.3%) T1DM, 13 (23.2%) T2DM, 2 (3.6%) GDM, 5 (8.9%) newly diagnosed	NA	NA	NA	NA	30.6% stillbirths
2022								
South Africa								
Guo	8 Cases, 27, 30, 35, 32, 28, 34, 29, 25 (?mean)	25, 32,15,33,18,30,32,12 wk	GDM/Overt diabetes	Not monitoring blood glucose, no antenatal care	GI symptoms, tachypnoea, hyperventilating, dehydration, tachycardia, agitated, weakness, osmotic symptoms, foetal tachycardia	NA	1× PIH	2× stillbirth, 1× abortion, 5 healthy babies
2008								
China								
Montero	20 Cases, mean age 25	24.2 in the group with live births and 30.9 in the group with foetal death	T1DM	Infection (4)	Polyuria, polydipsia, nausea, vomiting, malaise, abdominal pain, headache, chest pain, seizures.	IV insulin and fluids; one patient received bicarbonate (pH < 7.05)	All fully recovered	65% live foetus
1993				Alcohol/drug abusers (2) Tongue				35% foetal death
USA				Laceration and starvation (1)				
				Poor compliances (7)				
				New onset of diabetes (8)				

### Studies with individual patient data (case reports and case series)

In our review of case reports and case series with individual patient data, we identified a total of 74 cases of DKA during pregnancy. Among these, 1 patient experienced a recurrence of DKA during the same pregnancy, and 2 patients had recurrences during subsequent pregnancies.

Mean age at diagnosis was 28.8 years and average time of gestation at diagnosis was 29.5 weeks. The majority of women had type 1 diabetes (T1DM) (45.9%; 34/74) and a fraction had fulminant T1DM (12.1%, 9/74). The second commonest type of diabetes was GDM (40.5%, 30/74). The remaining cases were type 2 diabetes (T2DM) (9.5%, 7/74), late onset diabetes (1.4%, 1/74), while in 2 cases the type was not specified and was reported as insulin dependent diabetes (2.7%, 2/74). The majority had classic DKA (70.3%, 52/74) and almost one-third of cases developed EDKA (29.7%, 22/74). In the GDM group, 40% (12/30) had EDKA and the remaining 60% (18/30) classic DKA. Parity was reported for 53 cases ranging from first to ninth pregnancy. Ethnicity data were available in 44 cases of whom 40.9% were Asian (18/44), 29.5% Caucasian (13/44), 20.8% African (9/44), 4.6% Latin American (2/44) and 4.6% Middle Eastern (2/44).

Most common trigger factor included infection (28%, 21/74), followed by poor adherence to treatment (13.5%, 10/74), treatment with steroids (12.1%, 9/74), new diagnosis (5%, 4/74), pump failure (4%, 3/74), hyperemesis (2.7%, 2/74), reduced carb intake (2.7%, 2/74), dehydration/long haul travel (1.3%, 1/74), pre-eclampsia (1.3%, 1/74) and labour (1.3%, 1/74). In almost one-third of the cases (29%, 20/74), a trigger factor was not identified or reported.

The most reported symptoms were nausea (32.4%, 24/74) and vomiting (32.4%, 24/74), osmotic symptoms (21.6%, 16/74), abdominal pain (20.2%, 15/74), shortness of breath (18.9%, 14/74) and fatigue (9.4%, 7/74). Intravenous insulin and fluids were used in all cases.

The vast majority (98.9%, 73/74) eventually fully recovered. Two women required mechanical ventilation (2.7%, 2/74) and 1 patient was on non-invasive ventilation (1.3%, 1/74). Death following acute respiratory distress syndrome and organ failure was reported in 1 case (1.3%, 1/74). Four patients had concurrent pre-eclampsia (5.4%, 4/74). One patient had a total hysterectomy due to stitch and tubo-ovarian abscess (1.3%, 1/74). Data on mode of delivery was available in 60 cases; vaginal birth was the most common (38.4%, 28/74) followed by emergency caesarean section (17.8%, 13/74), elective caesarean section (8.2%, 6/74) and operative vaginal birth (1.4%, 1/74). Induction of labour was also used in a small number of cases (16.4%, 12/74).

Data on neonatal outcomes were available for 71 cases. Intrauterine death / stillbirth was reported in one-third of cases (35.2%, 25/71) of whom one was a twin pregnancy. Mean gestational age when foetal death occurred was 30 weeks. Termination of pregnancy was decided in 1 case (1.4%, 1/74). Information on the gestational age at delivery was reported in 58 cases with the majority of patients delivering preterm (63.7%, 37/58) and the remaining delivering at term (36.2%, 21/58). Data on foetal growth was available in 46 cases of whom 6 babies were large for gestational age (13%, 6/46). Data on foetal glucose readings was not consistently provided. Information on amniotic fluid volume was provided in 26 cases of whom 5 (19.2%, 5/26) had polyhydramnios.

### Studies without individual patient data (case series and observational studies)

We identified 9 case series without individual patient data.^3,66-73^ Schneider et al reported 4 cases of DKA on the background of GDM with mean gestational age at 29 + 1 weeks. The most common precipitating factor was infection. There were no maternal deaths, but 1 foetal demise was reported. Cullen et al reported 11 cases with pre-existing type 1 or 2 diabetes with 1 foetal demise and 4 macrosomic infants. Similarly, Kilvert et al published 10 cases, of which 9 had pre-existing diabetes and 1 pre-diabetes; 6 of the 8 patients treated with insulin at the time of the ketoacidosis had 7 further episodes of ketoacidosis and at least 11 admissions for severe hyperglycaemia either before or after DKA. There were 2 foetal deaths. Dhanasekaran et al reported 71 cases, 48 with T1DM (82.8%) and 23 with T2DM (17.2%); foetal demise occurred in 10 pregnancies (6 miscarriages and 4 stillbirths) and macrosomic infants in 16 cases (33.3%).

In a case series with fulminant diabetes, foetal demise occurred in 21 out of the 23 cases (91.3%) (Liu et al). No maternal or foetal deaths were reported by Bryant et al whose cohort consisted of 33 women with the majority (67%) having T1DM. On the other hand, Maseko et al reported 56 cases with 30.6% stillbirth rate. Guo et al identified 8 DKA events out of 90 patients with GDM/overt diabetes with 2 stillbirths and 1 spontaneous abortion. Finally, Montero et al reported high frequency of foetal death in 35% of the cohort which consisted of 20 women with T1DM.

### Quality of the studies

Regarding case reports and series quality assessment revealed that most studies were graded either as of good or moderate quality. Details on demographic characteristics and interventions of the included cases was not sufficiently reported in 20 and 18 articles respectively. Only 1 study did attain a perfect score (Table S1).

Among studies without individual patient data, only 1 study was considered to have a high risk of bias. The rest were either low risk of bias (4/9) or medium risk of bias (4/9). The most frequently missing information was the prospective sample size calculation, which was absent in all studies (0/9) (Table S2).

## Discussion

In this systematic review, 74 cases of DKA during pregnancy were identified, predominantly in women with GDM or T1DM, most frequently occurring during the third trimester. Typical symptoms included nausea, vomiting, and osmotic symptoms (polyuria, polydipsia) preceding the onset of DKA. Although classic DKA with hyperglycaemia was the most common presentation, almost 30% of cases were EDKA, requiring high clinical suspicion for prompt diagnosis and management. EDKA was common in patients with gestational diabetes (40%). All but 1 woman fully recovered. However, 25.6% of the cases were associated with foetal demise. Trigger factors included infections (with several cases published during the COVID-19 pandemic), poor adherence to insulin therapy, and the use of steroids for lung maturation.

### Results in the context of the literature

With the ultimate goal of providing adequate nutrition to the foetus, insulin resistance increases during pregnancy due to rising hormones such as progesterone, peaking during the second and third trimesters. As resistance increases with gestational age, insulin production is enhanced to compensate for the higher requirements. This is achieved through pancreatic beta cell hyperplasia. Additionally, a relative state of starvation and hypoglycaemia characterises pregnancy, leading to increased lipolysis and subsequent ketogenesis, which further supports the nutritional needs of the foetus.^74^ On the background of a precipitating factor such as an infection, decompensation and subsequent diabetic ketoacidosis might occur.^75^ Therefore, pregnancy is a ketogenic state that can progress to diabetic ketoacidosis when a trigger factor enhances ketogenesis against a background of relative insulin deficiency.^76,77^ It is worth noting that since respiratory alkalosis is also seen in pregnancy, baseline bicarbonate levels are already lower due to compensation. The above also explain why DKA most commonly presents in late pregnancy; This was also evident in our study with most reported cases presenting in women during the third trimester. However, since it can still occur early in pregnancy, it’s important to maintain a low threshold for ketone testing when women present with relevant symptoms even in the absence of previous diagnosis of diabetes.^24^

EDKA presents more frequently during pregnancy due to increased expression of placental glucose transporters, which aim to provide nutrition to the foetal-placental unit and lead to lower blood glucose levels. Interestingly, these receptors are higher in people treated with insulin. Moreover, increased plasma volume is seen in pregnancy leading to haemodilution and therefore normal blood glucose levels. EDKA is equally detrimental as classic DKA in pregnancy and can result in cardiac arrhythmias secondary to electrolyte imbalances,^20^ myocardial infarction, and cardiogenic shock secondary to acidosis requiring ITU admission. There is a higher risk of recurrent decelerations, foetal hypoxia and mortality before and during delivery of the foetus. Ketones can also cross the placental barrier causing intellectual disability, encephalomalacia, cleft lip, cleft palate, and neural tube defects.^78^ As there is an inverse relationship between maternal ketone levels and peripartum/neonatal outcomes, early diagnosis and treatment of classic and EDKA is crucial. It’s worth noting that in some cases, recurrent episodes of DKA were reported during the same or subsequent pregnancies. Multiple episodes of DKA during pregnancy pose a high risk to the foetus and increase the chances of foetal complications and demise.^79-81^ Causes of recurrent DKA include omission of insulin and poor adherence to treatment.^82^ Therefore, patients after the first DKA episode require increased attention.

A notable observation is that the most frequently reported ethnic background in our study was Asian, which is partly explained by the existing data on a higher prevalence of GDM in women of South Asian and South East Asian decent compared to Caucasian, African-American and Hispanic.^83^ Another important observation is the high percentage of women with GDM. Previous studies conducted in 2019 to 2022, such as a case-control study in the United Kingdom, have indicated that the majority of DKA cases occurred in individuals with T1DM, while GDM was still recognised as a risk factor. However, the high percentage of GDM cases in our study suggests that the population is changing and this is likely due to the increasing prevalence of obesity linked to insulin resistance.^84^ Therefore, this emergency condition can be expected in women regardless of the underlying type of diabetes.^24,79^ Another interesting finding is the frequency of macrosomia which was 13% based on the available information for 46 cases and is consistent with the reported worldwide prevalence.

### Strengths and limitations

Our study constitutes the first systematic review providing an overview of the cases of DKA during pregnancy. This research provides a detailed synthesis of existing literature, incorporating quality assessment of the included studies. Despite the comprehensive nature of our review, it is imperative to acknowledge several inherent limitations. A primary limitation arises from the inclusion of low-quality case reports and case series, which may compromise the validity and generalisability of our conclusions. These types of studies are prone to biases, such as overinterpretation and selection bias, potentially skewing the results. Therefore, while the findings reported herein are noteworthy, they may not fully capture the variety associated with DKA during pregnancy regarding presentation, management and outcomes. To establish a stronger body of evidence, further insights are required ideally from prospectively design studies.

## Conclusion

In this systematic review, DKA during pregnancy most commonly presented in women with T1DM or GDM. It was often triggered by infections, suboptimal adherence to treatment, and steroid administration. DKA can be life-threatening for the mother and can also lead to foetal demise which occurred in around one-fourth of the cases in this cohort. These findings underscore the necessity for clinicians to consider the possibility of DKA in any deteriorating pregnant woman. Early recognition, immediate hospitalisation and aggressive treatment remain the mainstay of DKA management.

It is important to recognise that pregnancy induces a diabetogenic state to facilitate the delivery of glucose to the foetus, ensuring proper growth. DKA during pregnancy is not exclusive to T1DM but also affects women with GDM and T2DM. Ongoing patient education on sick day rules and the importance of adherence to insulin therapy, increased awareness among both patients and clinicians, and careful glucose monitoring during steroid administration are all crucial in preventing, or at the very least effectively managing, diabetic emergencies during pregnancy. This approach is essential to reducing adverse outcomes associated with DKA.

## Supplemental Material

sj-docx-1-end-10.1177_11795514241312849 – Supplemental material for Diabetic Ketoacidosis in Pregnancy: A Systematic Review of the Reported Cases Supplemental material, sj-docx-1-end-10.1177_11795514241312849 for Diabetic Ketoacidosis in Pregnancy: A Systematic Review of the Reported Cases by Dimitra Stathi, Florence Ning Lee, Mili Dhar, Stergios Bobotis, Elisavet Arsenaki, Taruna Agrawal, Konstantinos Katsikas Triantafyllidis and Konstantinos S Kechagias in Clinical Medicine Insights: Endocrinology and Diabetes

## Declarations

**Ethics Approval and Patient Consent:** Not applicable. Ethical approval was not required for this review.

**Consent for Publication:** Not applicable.

**Availability of Data and Materials:** The authors confirm that the data supporting the findings of this study are available within the article and its supplementary materials.

**Transparency Declaration:** The lead/corresponding author (KSK) affirms that this manuscript is an honest, accurate, and transparent account of the study being reported; that no important aspects of the study have been omitted; and that any discrepancies from the study as planned have been explained. All claims expressed in this article are solely those of the authors and do not necessarily represent those of their affiliated organisations.
