# Supplementary material for: Diabetic Ketoacidosis in Pregnancy: A Systematic Review of the Reported Cases
Source: Clin Med Insights Endocrinol Diabetes. 2025 Jan 15;18:11795514241312849. doi: 10.1177/11795514241312849 (PMC11733887; doi:10.1177/11795514241312849)
Supplement: sj-docx-1-end-10.1177_11795514241312849 – Supplemental material for Diabetic Ketoacidosis in Pregnancy: A Systematic Review of the Reported Cases [file sj-docx-1-end-10.1177_11795514241312849.docx]

**Supplementary material**

**Table S1.** Quality assessment of the included studies (studies with individual patient data)

| **Author, year** | **Q1** | **Q2** | **Q3** | **Q4** | **Q5** | **Q6** | **Q7** | **Q8** |
| --- | --- | --- | --- | --- | --- | --- | --- | --- |
| Ali, 2023 | Ø | ● | ● | ● | ● | ● | **○** | ● |
| Stamatiades, 2023 | ● | ● | ● | ● | ● | ○ | ○ | ● |
| Bereda, 2022 | ● | ● | ● | ● | ● | ● | ○ | ● |
| Harman, 2022 | ● | ● | ● | ● | ● | ● | ● | ● |
| Velasco, 2022 | Ø | ○ | ○ | ● | ● | ● | ● | ○ |
| Wazir, 2022 | ● | ● | ● | ● | ● | ● | ○ | ● |
| Aminimoghaddam, 2021 | Ø | Ø | Ø | ● | ● | ● | ● | ● |
| Dargel, 2021 | ● | ● | ● | ● | ● | ● | ● | ● |
| Eshkoli, 2021 | Ø | ○ | ● | Ø | ● | ● | ○ | ● |
| Smati, 2021 | ● | ○ | ● | ○ | ● | ○ | ○ | ● |
| Muppidi, 2020 | Ø | Ø | ● | Ø | ● | ● | ○ | ● |
| Yaron, 2020 | Ø | ○ | ● | ● | Ø | ● | ● | ● |
| Jaber, 2019 | Ø | Ø | Ø | Ø | ● | ● | ● | ● |
| de Alencar, 2019 | Ø | Ø | ● | ● | ● | ● | ● | ● |
| Hayakawa, 2019 | ● | ● | ● | ● | ● | ○ | ○ | ● |
| Lucero, 2018 | ● | ○ | Ø | ● | Ø | Ø | ○ | ● |
| Cardonell, 2016 | Ø | ● | ● | ● | ● | ● | ● | ● |
| Farrant, 2016 | ● | ● | ● | ● | ● | ○ | ● | ● |
| Cen, 2014 | Ø | Ø | ● | ● | ● | ○ | ● | ● |
| Dorey, 2014 | Ø | Ø | ● | ● | ● | ● | ● | ● |
| Graham, 2014 | ● | ● | ● | ● | ● | ● | ● | ● |
| Himuro, 2014 | ● | ● | Ø | ● | ● | ● | ○ | ● |
| Melville, 2014 | ● | ○ | ● | ● | ● | ● | ● | ● |
| Yan, 2013 | Ø | ○ | ● | ○ | Ø | Ø | ● | ● |
| Dharbamulla, 2012 | ● | ● | ● | ● | ● | ● | ○ | ● |
| Kim, 2012 | ● | ● | ● | ● | ● | ○ | ● | ● |
| Napoli, 2011 | ● | ● | ● | ● | ● | ● | ○ | ● |
| Pinto, 2011 | ● | ○ | Ø | Ø | Ø | Ø | ○ | ● |
| Lee, 2010 | ● | ● | ● | ● | ● | Ø | ○ | ● |
| Tan, 2010 | ● | ● | ● | ● | Ø | ● | ● | ● |
| Franke, 2009 | ● | ○ | ● | ● | ● | ● | ○ | ● |
| Chico, 2008 | ● | ● | ● | ○ | ● | ● | ● | ● |
| Oliver, 2007 | ● | Ø | ● | ● | Ø | ● | ○ | ● |
| Tarif, 2007 | Ø | ○ | ● | ● | Ø | ● | ○ | ● |
| Yamamoto, 2007 | ● | ● | ● | ● | ● | ● | ● | ● |
| Kamalakanan, 2003 | Ø | Ø | ● | ● | ● | ○ | ● | ● |
| Deepak, 2002 | Ø | Ø | ● | ● | ● | ● | ● | ● |
| Inagaki, 2002 | ● | ○ | ● | ● | Ø | ● | ● | ● |
| Otsubo, 2002 | Ø | ○ | ● | ● | Ø | Ø | ● | ● |
| Trivedi, 2002 | Ø | Ø | ● | ● | ● | ● | ○ | ● |
| O'Shaughnessy, 1999 | ● | ● | ● | ● | ● | ● | ○ | ● |
| Sasuga, 1999 | ● | ● | ● | ● | ● | ● | ● | ● |
| Pitteloud, 1998 | ● | ● | ● | ● | Ø | ○ | ○ | ● |
| Bedalov, 1997 | ● | Ø | ● | ● | ● | ● | ○ | ● |
| Ko, 1995 | ● | Ø | ● | ● | Ø | ● | ● | ● |
| Sills, 1994 | ● | Ø | ● | ● | Ø | ● | ● | ● |
| Abourizk, 1993 | ● | ● | ● | ● | Ø | ● | ● | ● |
| Lindenbaum, 1993 | ● | Ø | ● | ● | Ø | ● | ○ | ● |
| Phuapradit, 1993 | ● | ● | ● | ● | Ø | Ø | ● | ● |
| Maislos, 1992 | ● | Ø | ● | ● | Ø | ● | ● | ● |
| Clark, 1991 | Ø | ● | ● | ● | ● | ○ | ● | ● |
| Bernstein, 1990 | Ø | ○ | Ø | ● | ● | ● | ● | ● |
| Bertolino, 1990 | Ø | Ø | ● | ● | Ø | ● | ● | ● |
| Halpren, 1988 | ● | ● | ● | ● | ● | ● | ● | ● |
| Rhodes, 1984 | ● | Ø | ● | ● | ● | ○ | ○ | ● |
| Robertson, 1986 | ● | ● | ● | ● | Ø | ○ | ● | ● |
| Knowles, 1962 | ● | ● | ● | ● | ● | ● | ● | ● |

Q1: Patient demographics; Q2: Medical History; Q3: Presentation; Q4: Diagnostic tests; Q5: Interventions; Q6: Post-intervention condition; Q7: Adverse events; Q8: Takeaway lessons ● = 1; ○ = No; Ø =Unclear

**Table S2.** Quality assessment of the included studies based on the Methodological Index for Non-Randomized Studies (MINORS) tool (studies without individual patient data).

| **Author**  **Year** | **Aim** | **Inclusion of consecutive patients** | **Prospective collection of data** | **Endpoints appropriate to the aim of the study** | **Unbiased assessment of the study endpoint** | **Follow-up period appropriate to the aim of the study** | **Loss to follow up less than 5%** | **Prospective calculation of the study size** | **Adequate control group** | **Contemporary groups** | **Baseline equivalence of groups** | **Statistical analysis** | **Total** | **Risk of bias** |
| --- | --- | --- | --- | --- | --- | --- | --- | --- | --- | --- | --- | --- | --- | --- |
| Dhanasekaran  2022 | 1 | 2 | 1 | 2 | 2 | 2 | 2 | 0 | NA | NA | NA | NA | 12/16 | Low |
| Maseko  2022 | 2 | 2 | 1 | 2 | 2 | 2 | 1 | 0 | NA | NA | NA | NA | 11/16 | Low |
| Liu  2018 | 1 | 2 | 1 | 2 | 2 | 0 | 2 | 0 | 2 | 2 | 2 | 2 | 18/24 | Low |
| Bryant  2017 | 1 | 2 | 0 | 2 | 2 | 2 | 0 | 0 | NA | NA | NA | NA | 9/16 | Some concerns |
| Guo  2008 | 1 | 1 | 0 | 2 | 1 | 2 | 2 | 0 | 2 | 2 | 0 | 1 | 14/24 | Low |
| Schneider  2003 | 0 | 0 | 0 | 2 | 2 | 2 | 2 | 0 | 0 | 2 | 0 | 0 | 10/24 | High |
| Cullen  1996 | 1 | 2 | 0 | 1 | 2 | 2 | 0 | 0 | NA | NA | NA | NA | 8/16 | Some concerns |
| Kilvert  1993 | 1 | 2 | 0 | 1 | 2 | 2 | 1 | 0 | NA | NA | NA | NA | 9/16 | Some concerns |
| Montoro  1993 | 1 | 2 | 0 | 2 | 2 | 2 | 0 | 0 | NA | NA | NA | NA | 9/16 | Some concerns |
